# Global Research Trends in Orthobiological Treatments for Osteoarthritis: A Bibliometric Analysis

**DOI:** 10.3390/healthcare14162504

**Published:** 2026-08-12

**Authors:** Seçkin Özcan, Erdinç Genç

**Affiliations:** 1 Department of Orthopaedics and Traumatology, Faculty of Medicine, Yalova University, Yalova 77200, Turkey; 2 Department of Orthopaedics and Traumatology, Istanbul Medipol University, Istanbul 34810, Turkey; erdincgenc@hotmail.com

**Keywords:** osteoarthritis, orthobiologics, platelet-rich plasma, mesenchymal stem cells, bibliometric analysis, regenerative medicine

## Abstract

**Highlights:**

**What are the main findings?**

- Scientific production on orthobiological treatments for osteoarthritis has increased markedly since 2015, with China emerging as the leading contributor and mesenchymal stem cells (MSCs) and platelet-rich plasma (PRP) remaining the dominant research topics.
- Research trends have shifted toward next-generation regenerative strategies, including exosomes, extracellular vesicles, biomaterials, and controlled drug delivery systems, indicating a transition from conventional biologics to advanced regenerative platforms.

**What are the implications of the main findings?**

- Orthobiological research is evolving from symptom-oriented interventions toward disease-modifying and regenerative approaches, reflecting growing interest in cartilage repair and joint preservation.
- Emerging themes such as exosome-based therapies and bioengineered delivery systems may shape future osteoarthritis research and provide promising directions for translational and clinical applications.

**Abstract:**

**Background/Objectives:** Osteoarthritis (OA) is a common degenerative joint disease associated with pain, functional limitation, and reduced quality of life. Interest in orthobiological treatments, including platelet-rich plasma (PRP), mesenchymal stem cells (MSCs), bone marrow aspirate concentrate (BMAC), exosomes, and biomaterial-based approaches has increased markedly in recent years. This study was aimed at investigating the bibliometric characteristics, research trends, and future directions of scientific literature on orthobiological treatments in OA. **Methods:** Publications indexed in the Web of Science Core Collection (WoSCC) between 2000 and 2026 were searched on 3 June 2026 using keywords related to OA and orthobiological therapies. The WoSCC was selected because it provides standardized citation data and comprehensive coverage of high-impact scientific journals for bibliometric analyses. In the study, 7875 publications were included. Bibliometric analyses were performed using the Bibliometrix package and Biblioshiny interface. Publication trends, country and institutional contributions, influential publications, keyword patterns, trending topics, and thematic structures were analyzed. **Results:** Scientific production on orthobiological treatments in OA increased substantially, particularly after 2015, with an annual growth rate of 22.8%. China was the most productive country, whereas the United States had the highest scientific impact. The most productive institutions were mainly universities in China. The most frequent keywords were “osteoarthritis,” “mesenchymal stem cells,” “platelet-rich plasma,” “knee osteoarthritis,” and “cartilage.” Trend analyses indicated a shift from PRP- and stem cell-based applications toward emerging regenerative strategies, including exosomes, extracellular vesicles, biomaterials, and controlled drug delivery systems. Thematic mapping demonstrated that MSCs and cartilage regeneration formed the central research axis. **Conclusions:** While MSCs and PRP remain key research topics in orthobiological treatments for OA, exosomes, biomaterials, and smart drug delivery systems are attracting increasing attention as crucial areas for future research.

## 1. Introduction

Osteoarthritis (OA), a chronic, progressive, and multifactorial joint disease, affects millions of people globally. It is characterized by pain, loss of function, and decrease in quality of life [1]. OA is not only a significant cause of disability in individuals, but is also associated with increased morbidity and mortality [2]. Several biological and environmental factors such as genetic predisposition, excessive mechanical loading of the joints, obesity, and post-traumatic joint injuries play a role in the development of OA [3,4,5]. The incidence of osteoarthritis has increased significantly in recent years, especially due to the increase in the aging population and the prevalence of obesity, and the extension of life expectancy [6,7]. Today, OA, considered as one of the most important causes of pain and loss of function in older adults, poses a significant economic burden on healthcare systems [8]. Patient education, weight control, exercise programs, pharmacological agents, and surgical procedures are among the widely used methods in the treatment of osteoarthritis [4,5]. However, the vast majority of current treatment methods focus on symptom control and have limited effect on altering the biological processes of OA or reversing cartilage degeneration, which has significantly increased the interest in regenerative medicine and orthobiological approaches in recent years.

Orthobiological therapies are defined as innovative regenerative approaches in which biologically derived products are used in the treatment of musculoskeletal diseases [9]. Platelet-rich plasma (PRP), bone marrow aspirate concentrate (BMAC), mesenchymal stem cells (MSCs), stromal vascular fraction (SVF), and exosomes are among the most frequently investigated orthobiological applications in the treatment of osteoarthritis today [9,10]. The leading aim in these treatments is to reduce inflammation, promote tissue healing, and restore joint homeostasis [11]. In the last two decades, a remarkable increase has been observed in scientific publications on orthobiological applications [12]. Interest in orthobiological therapies has increased due to their potential to create disease-modifying effects in addition to the symptomatic benefits of conventional treatments [9,11]. After studies in which the focus is particularly on PRP and mesenchymal stem cell-based therapies, cell-free biological products and exosome-based approaches have come to the forefront in research in recent years [13,14]. However, the rapid growth in the literature makes it necessary to evaluate research trends, collaboration networks, and emerging research themes systematically.

Bibliometric analysis, a powerful method, allows quantitative evaluation of scientific production in a specific research field [15,16]. Thanks to this method, not only publication trends, influential authors, institutions, and countries can be determined, but also the intellectual structure and future directions of the research field can be revealed. Bibliometric analysis not only provides an overview of current knowledge for researchers but also contributes to the determination of priorities in future research. Although several bibliometric studies in which the focus is on specific treatment methods such as PRP, stem cells, BMAC, and hyaluronic acid in osteoarthritis have been published, there is no comprehensive bibliometric analysis that holistically reveals the knowledge structure and research trends of the field by analyzing different orthobiological agents under the same roof [10,17,18]. Although individual orthobiological therapies or specific aspects of regenerative medicine have been investigated in several bibliometric studies, a comprehensive bibliometric evaluation encompassing the full spectrum of orthobiological treatments for osteoarthritis is currently lacking. Moreover, rapidly emerging research areas such as exosomes, extracellular vesicles, biomaterials, and advanced drug delivery systems have not yet been systematically integrated into previous bibliometric analyses. Therefore, in this study, it is expected to provide a comprehensive global overview of the scientific landscape, to identify evolving research trends and collaboration patterns, and to highlight emerging research priorities to support future investigations and clinical translation in the field of orthobiological treatments for osteoarthritis. Therefore, in the current study, the aim was to investigate global research trends for orthobiological treatments in osteoarthritis using bibliometric methods, to reveal the knowledge structure of the field, and to identify areas for future research.

## 2. Materials and Methods

### 2.1. Study Design

The present study is a bibliometric and science mapping analysis conducted to investigate the structure, development, and research trends in the scientific literature on orthobiological treatments used in osteoarthritis. Since the study is based exclusively on the analysis of published bibliographic data, it does not require ethical committee approval.

### 2.2. Source of the Data and Search Strategy

Data for the bibliometric analysis were obtained from the Web of Science Core Collection (WoSCC) database, which is widely used in bibliometric studies because it indexes high-impact international journals and provides standardized citation data. The literature search was conducted on 3 June 2026. Therefore, publication data for 2026 represent only a partial publication year and should be interpreted accordingly. All indexes available in the Web of Science Core Collection were included in the search. The search strategy was designed to identify studies on orthobiological applications for osteoarthritis. A Topic Search (TS) was performed, in which orthobiologics-related terms were combined with osteoarthritis-related terms using the Boolean operator AND, while synonymous terms within each concept were connected using the Boolean operator OR. The exact search query was as follows:

No restriction was applied to the publication year, and all records indexed in the database were considered.

TS=(

“platelet-rich plasma”
OR PRP
OR “bone marrow aspirate concentrate”
OR BMAC
OR “mesenchymal stem cell*”
OR MSC
OR “stromal vascular fraction”
OR SVF
OR exosome*
OR “extracellular vesicle*”
OR “adipose-derived stem cell*”
)

AND

TS=(

osteoarthritis
OR “knee osteoarthritis”
OR gonarthrosis
)

### 2.3. Inclusion and Exclusion Criteria

Records were filtered using the Web of Science Core Collection filtering options before bibliometric analysis. Only English-language publications classified as Articles or Review Articles were included in the final dataset. Publications meeting the following criteria were included in the analysis:

- Studies published in English;
- Article and review publications;
- Studies directly related to osteoarthritis and orthobiological treatments.

The following publications were excluded from the study:

- Letters to the editor;
- Conference proceedings;
- Meeting abstracts;
- Duplicate versions of early access records;
- Correction articles;
- Book chapters;
- Retracted publications.

**Figure 1:**
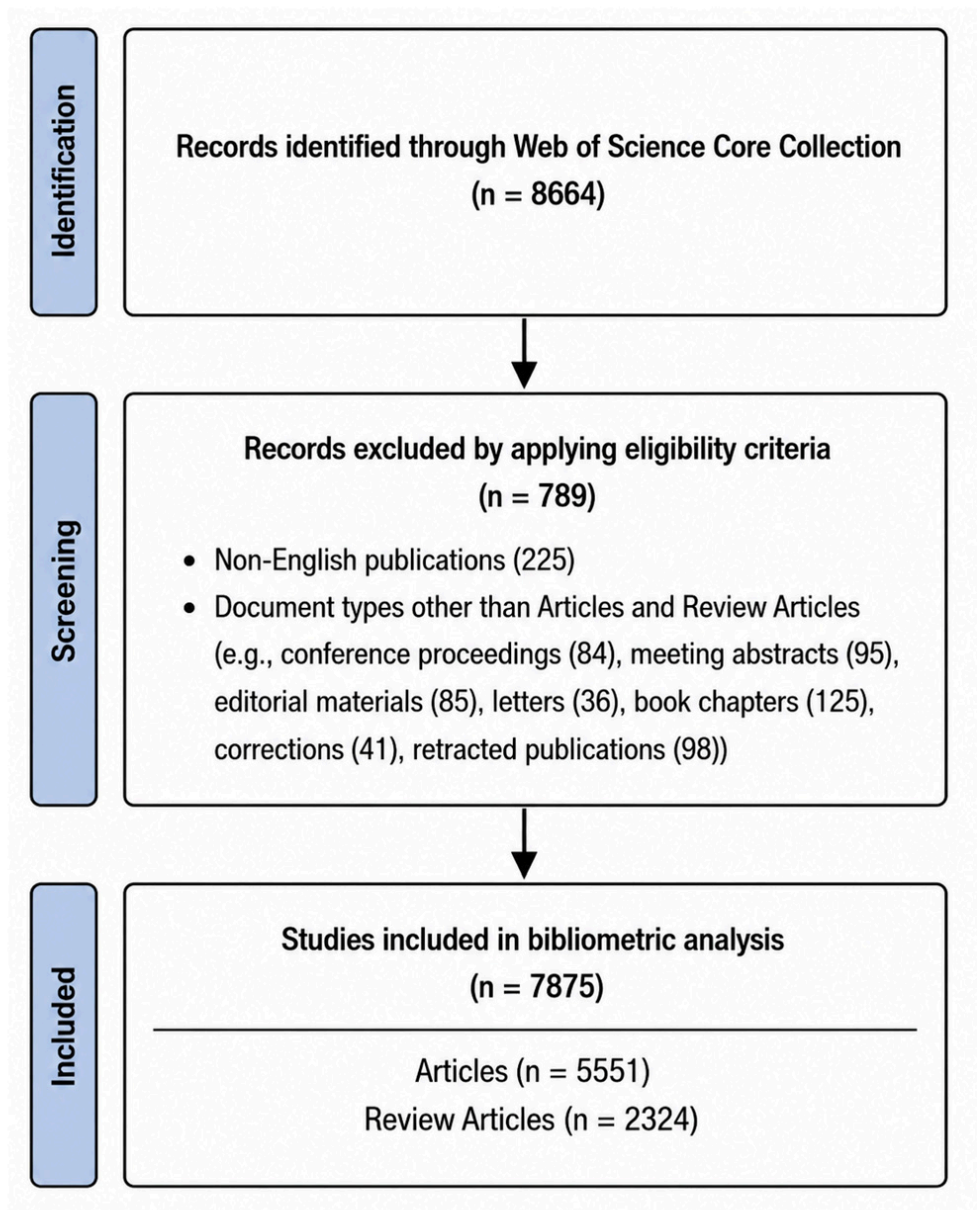
PRISMA 2020 flow diagram of the literature search and study selection process.

These exclusions were applied using the predefined Web of Science filtering options prior to data export. The publication selection process was reported in accordance with the PRISMA 2020 statement (Figure 1).

### 2.4. Data Extraction and Preparation Process

All records were exported from the Web of Science database in “Full Record and Cited References” format. In the resulting dataset, publication year, author information, institutions, countries, journals, keywords, citation counts, and reference information were included. Before the analysis was conducted, the dataset was checked for duplicate records, and necessary data cleaning operations were performed. Singular and plural uses of keywords, abbreviations, acronyms, and synonyms were reviewed to ensure standardization.

### 2.5. Bibliometric Analysis and Science Mapping

Bibliometric analyses and visualizations were performed using the Bibliometrix package (version 4.3.2) and the Biblioshiny interface (version 4.3.2) running in R software (version 4.5.0) [15].

In the performance analyses, the following were included:

- Publication output by year;
- Most prolific authors;
- Most prolific institutions;
- Most prolific countries;
- Most influential journals;
- Most cited publications.

Keyword co-occurrence analyses were conducted to reveal the conceptual structure of the research field. Within the research field, the main themes and focuses in research were analyzed by using author keywords and Keywords Plus data. Trend topic and thematic map analyses were performed to identify changing trends in the research field. Thanks to these analyses, the development of research themes and the structure of research clusters related to orthobiological treatments in osteoarthritis were analyzed. In addition, the scientific productivity of different countries was analyzed to assess the global research structure in the field. The results were presented in tables and visual information maps.

## 3. Results

### 3.1. General Bibliometric Characteristics

In the analysis, 7875 publications published between 2000 and 2026 in 1254 different journals were included. The number of cited references extracted from the included publications was 198,048. The dataset involved 26,934 researchers. The average number of co-authors per publication was 6.82, and the international co-authorship rate was 21.33%. The average age of the publications was 5.65 years, and the average number of citations per publication was 35.45. The annual growth rate of the literature was 22.8%. The dataset included 9397 author keywords and 7450 Keywords Plus terms. Of the included publications, 5551 were original research articles, and 2324 were review articles (Table 1).

### 3.2. Scientific Production Trends by Years

**Figure 2:**
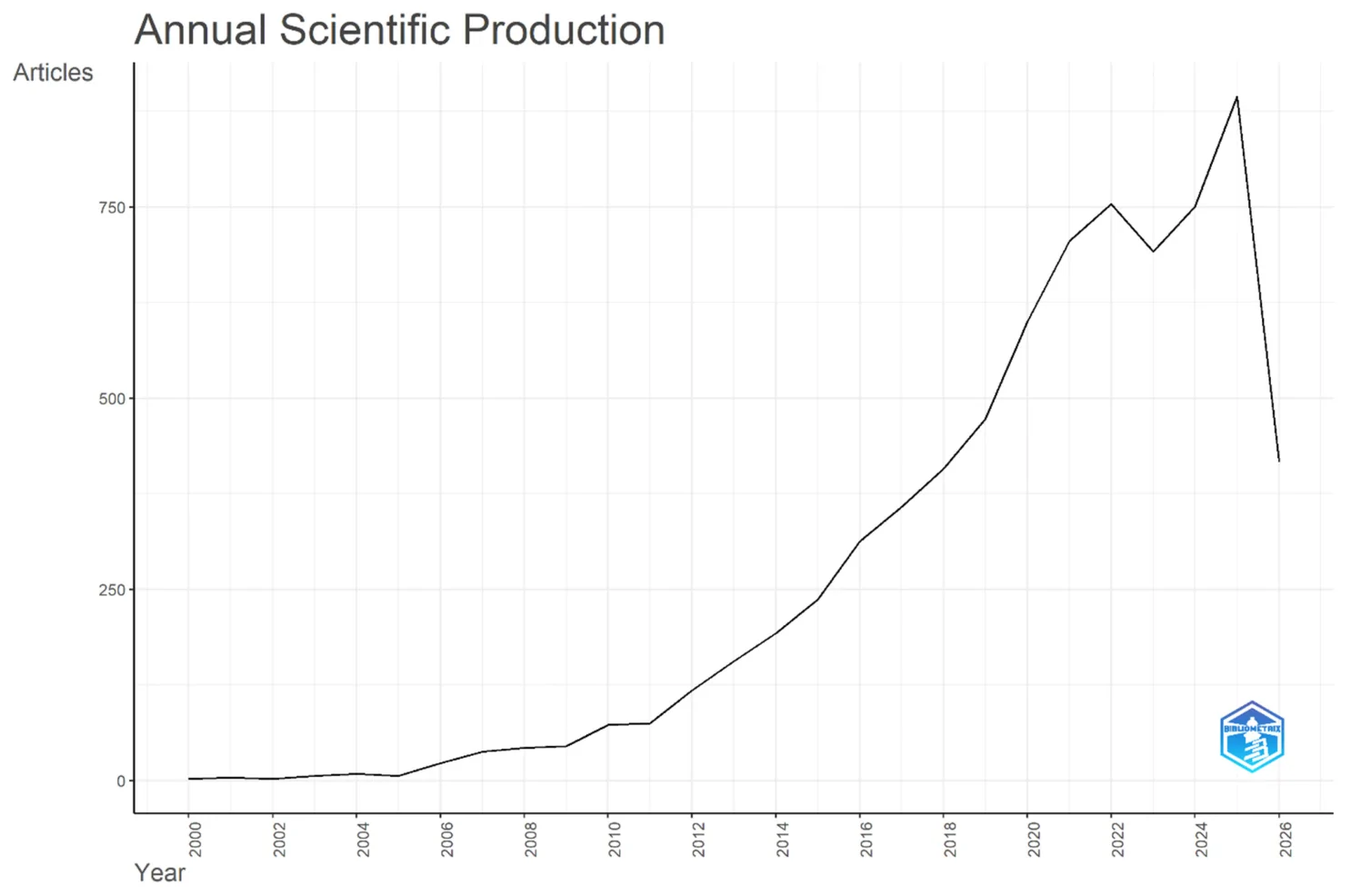
Scientific output of publications on orthobiological treatments in osteoarthritis by year (2000–2026). Note: Publication data for 2026 represent records indexed up to 3 June 2026, and therefore correspond to a partial publication year.

During the period analyzed in the present study (2000–2026), a steady increase was observed in the number of publications conducted on orthobiological treatments in osteoarthritis. In particular, a significant acceleration in publication production occurred after 2015, and the field has grown rapidly in recent years (Figure 2). Because the search was conducted on 3 June 2026, publication data for 2026 are incomplete and should not be directly compared with those of previous full calendar years.

### 3.3. Sources and Journals

The analysis of the distribution of the publications revealed that the field had a multidisciplinary structure. The journal with the most publications was the *International Journal of Molecular Sciences* (n = 238), followed by *Osteoarthritis and Cartilage* (n = 195), *American Journal of Sports Medicine* (n = 140), *Stem Cell Research & Therapy* (n = 123), and *Arthroscopy-The Journal of Arthroscopic and Related Surgery* (n = 92). *Osteoarthritis and Cartilage*, *American Journal of Sports Medicine*, and *Journal of Orthopaedic Research*, the high-impact journals in the field of orthopedics and musculoskeletal systems, ranked at the top, indicating that the subject received significant attention in the fields of clinical orthopedics and regenerative medicine (Table 2). This distribution indicates that orthobiological research has become highly interdisciplinary, attracting interest from orthopedics, regenerative medicine, tissue engineering, and molecular biology.

### 3.4. Author Analysis

The analysis of author productivity revealed that Filardo G. was the most prolific researcher (n = 87), followed by Wang Y. (n = 81), Liu Y. (n = 71), Wang J. (n = 69), and Zhang Y. (n = 64). Most of the top-ranking researchers are of Asian or European origin, indicating that the field is developing at an international level (Table 2). This distribution suggests that orthobiological research is driven by internationally recognized research groups with strong expertise in regenerative medicine and translational musculoskeletal research.

### 3.5. Institutional Productivity

The analysis of institutional productivity revealed a significant dominance of China-based universities. Sichuan University, with 347 publications, ranked first. Shanghai Jiao Tong University, with 326 publications, and Zhejiang University, with 305 publications, ranked second and third, respectively. The University of California System with 200 publications, Peking University with 189 publications, and Harvard University with 175 publications were among the other highly prolific institutions. The fact that a significant portion of the top ten institutions are of Chinese origin indicates that orthobiological research has been predominantly concentrated in Asian countries in recent years (Table 2). These findings indicate that institutional leadership in orthobiological research is increasingly concentrated in Chinese academic centers, reflecting sustained national investment in regenerative medicine and musculoskeletal research.

### 3.6. Scientific Production by Country

According to the analysis of scientific production on a country basis, the top five countries were as follows: while China was the most prolific country in the field with 8703 publications, the United States ranked second with 4989 publications, Italy ranked third with 1642, South Korea ranked fourth with 1038 publications, and Japan ranked fifth with 959 publications. According to the total number of citations, China with 70,316 citations and the United States with 60,817 citations stood out as the most influential countries in the field. In terms of average citations per article, the United States with 45.6 citations per article and South Korea with 42.7 citations per article were among the countries with a high level of impact (Table 3). The analysis of the network of cooperation between countries demonstrated that there were intensive international research collaborations, especially between North American, European, and East Asian countries.

### 3.7. Most Cited Publications

The study with the highest total number of citations is Hunziker et al.’s study published in 2002 (1619 citations), followed by Makris et al.’s review study published in 2015 (1061 citations) and Goldring et al.’s study published in 2007 (1051 citations). It is noteworthy that Yao et al.’s study, although it was published in 2023, reached 956 citations in a short time. These results indicate that fundamental studies on osteoarthritis biology, cartilage regeneration, and orthobiological treatments form the intellectual foundation of the field (Table 4).

### 3.8. Trend Topic Analysis

**Figure 3:**
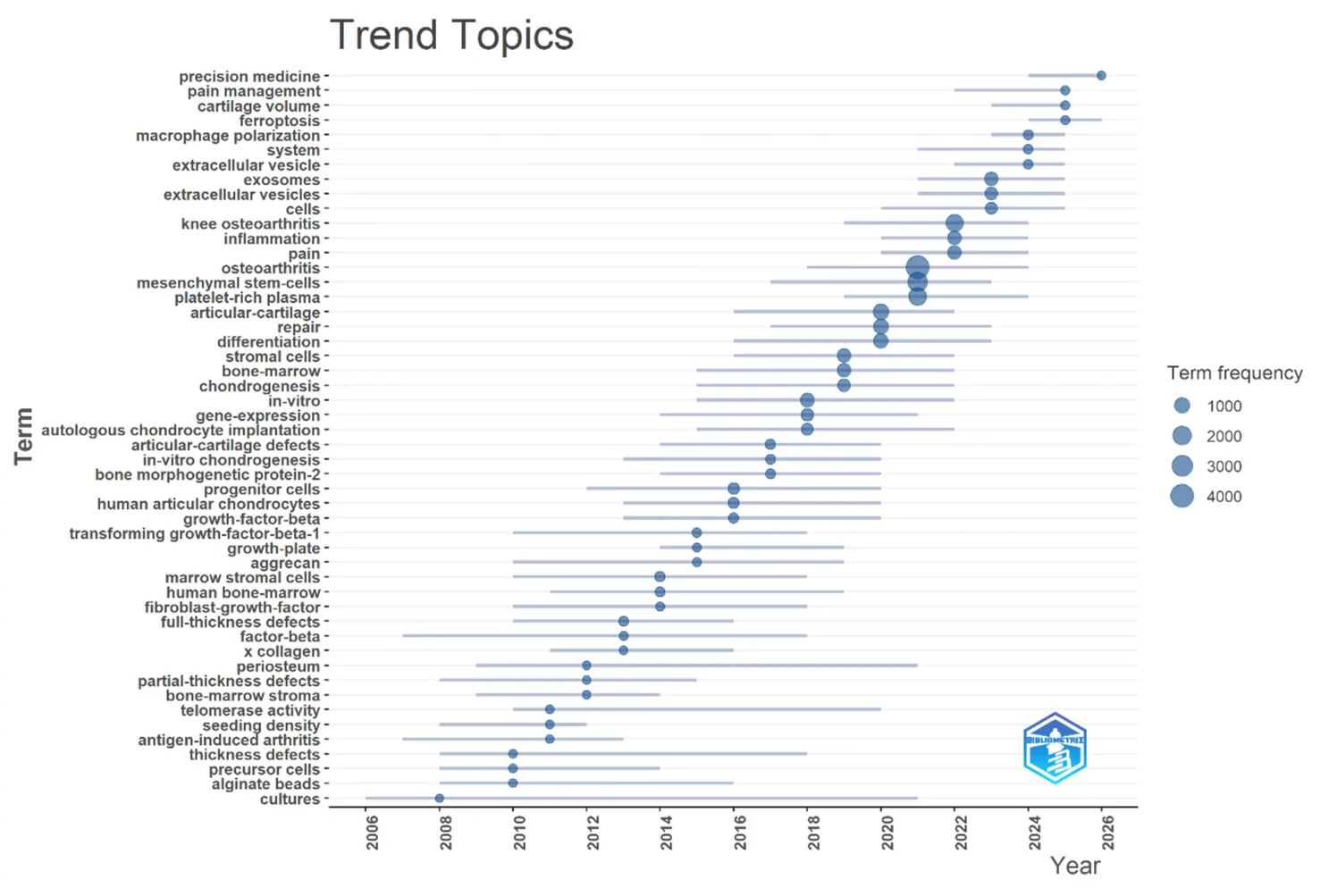
Temporal evolution of research topics for orthobiological treatments in osteoarthritis (2006–2026). Note: Publication data for 2026 represent records indexed up to 3 June 2026, and therefore correspond to a partial publication year.

The analysis of research trends over time revealed that in early studies, the focus was on culture systems, cartilage defects, growth factors, and bone marrow stromal cells. In the middle period, concepts such as chondrogenesis, autologous chondrocyte implantation, mesenchymal stem cells, and osteoarthritis came to the forefront. In recent years, topics such as exosomes, extracellular vesicles, macrophage polarization, precision medicine, pain management, and cartilage volume have emerged as research fields (Figure 3). Basic science-oriented topics such as chondrogenesis, growth factors, and bone marrow stromal cells were at the forefront in the early period. However, in the following years, an increase has been observed in mesenchymal stem cells and cartilage repair studies. Recently, PRP, osteoarthritis, inflammation, exosomes, and extracellular vesicles have emerged as the fastest-growing research fields.

### 3.9. Thematic Structure Analysis

**Figure 4:**
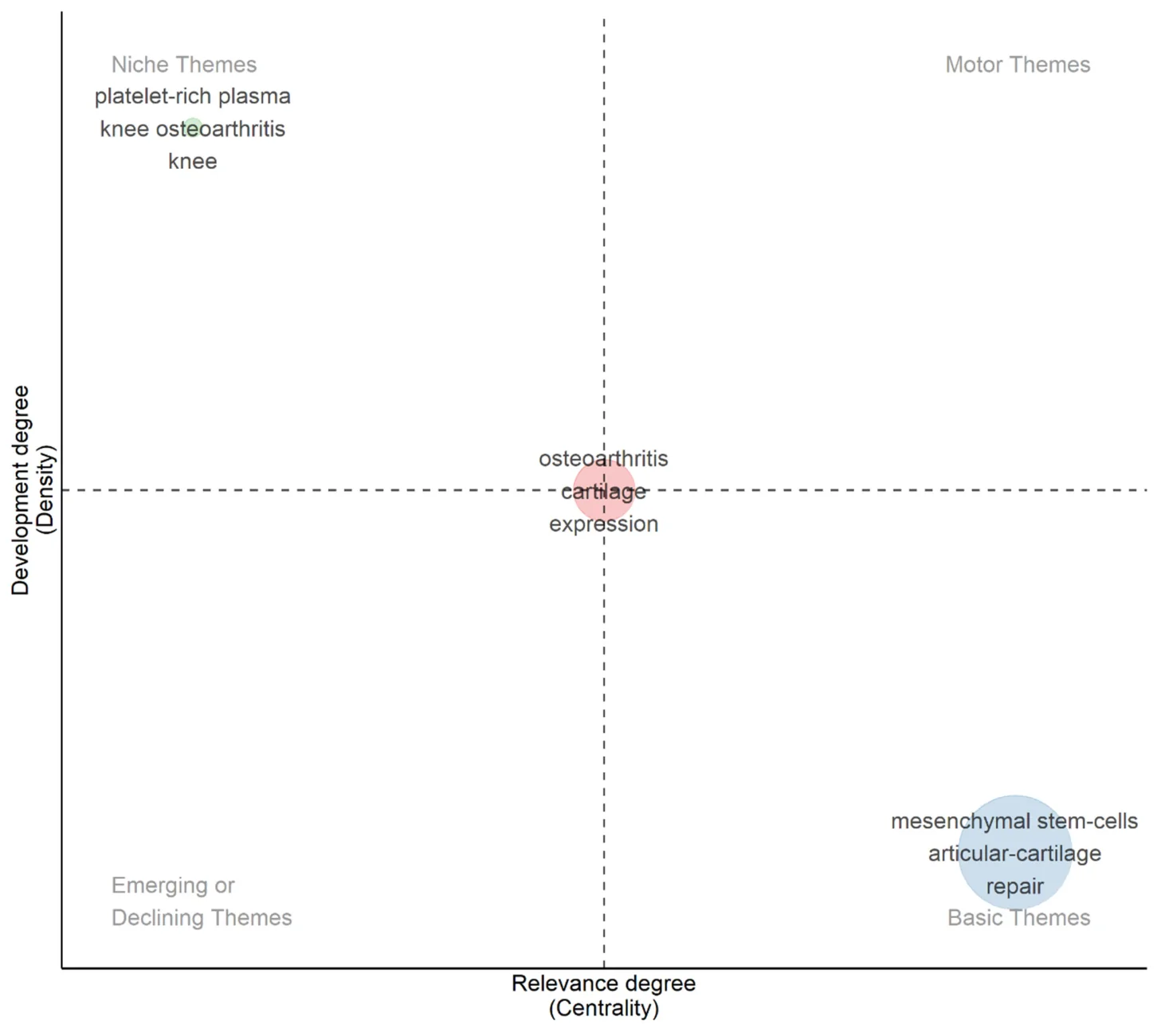
Distribution of research themes for orthobiological treatments in osteoarthritis according to their centrality and intensity levels.

The thematic map demonstrated that orthobiological research in osteoarthritis is primarily organized around two dominant thematic domains: mesenchymal stem cell-based cartilage regeneration and PRP-centered clinical applications (Figure 4). The first thematic domain encompasses regenerative medicine studies focusing on mesenchymal stem cells, articular cartilage, tissue repair, and regeneration processes. The second thematic domain comprises clinical application studies centered on platelet-rich plasma (PRP), hyaluronic acid, intra-articular injections, and knee osteoarthritis. In addition, osteoarthritis, cartilage biology, gene expression, and inflammation emerged as foundational concepts linking these domains and forming the conceptual core of the field.

The thematic map further revealed that mesenchymal stem cells, articular cartilage, and tissue repair were positioned within the basic themes quadrant, indicating their central role in shaping the intellectual structure of the field. In contrast, PRP and knee osteoarthritis clusters exhibited high density and developmental maturity but lower centrality, suggesting that they represent specialized yet well-established research areas. Osteoarthritis and cartilage biology occupied a central position within the thematic structure, serving as key conceptual bridges between different research domains (Figure 4).

### 3.10. Keyword Analysis

**Figure 5:**
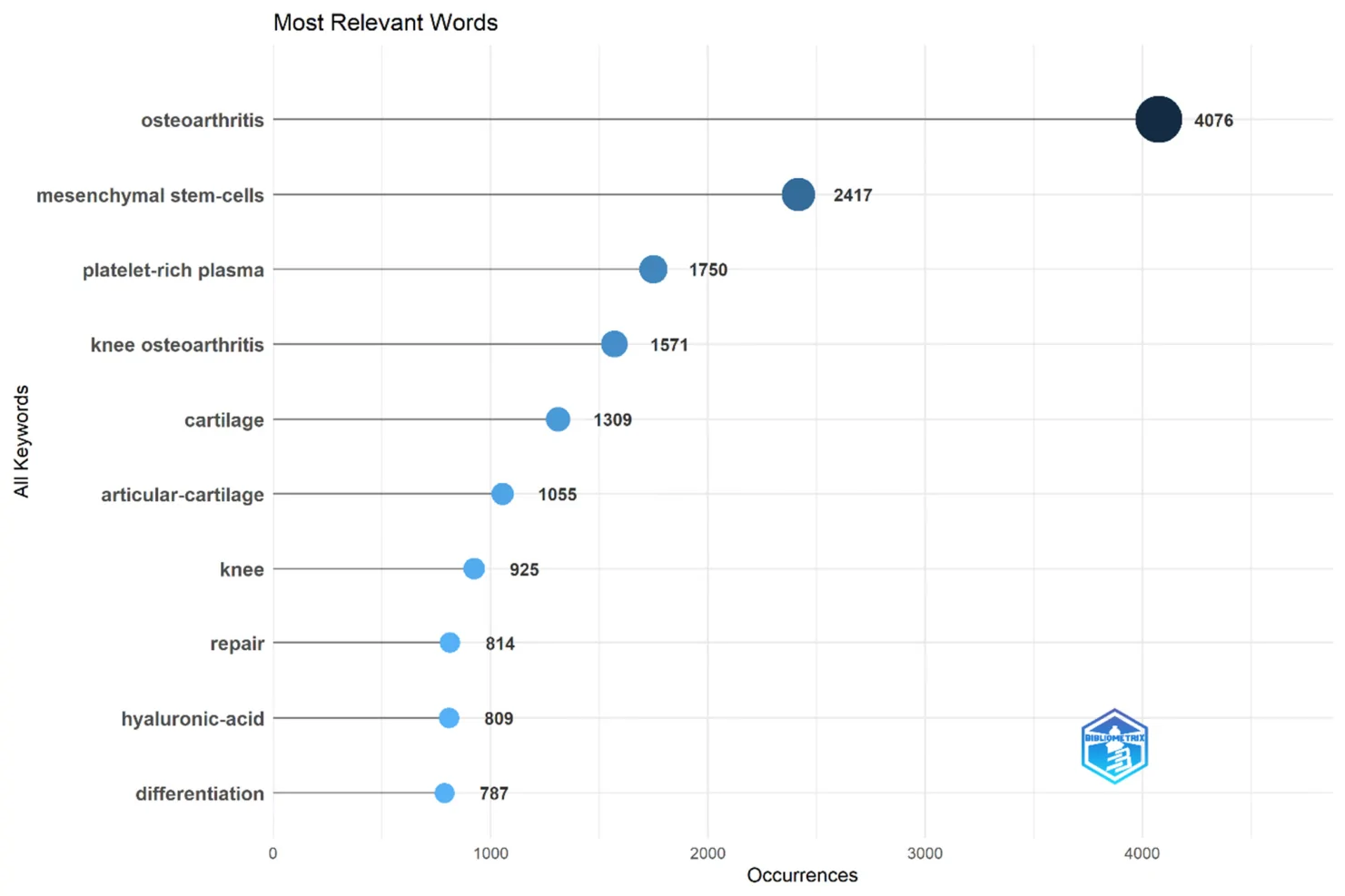
The most frequently used keywords in orthobiological research on osteoarthritis. Bubble size and color intensity represent keyword frequency, with larger and darker bubbles indicating higher numbers of occurrences.

Keyword analysis revealed that the most frequently used terms were osteoarthritis (n = 4076), mesenchymal stem cells (n = 2417), platelet-rich plasma (n = 1750), knee osteoarthritis (n = 1571), cartilage (n = 1309), and articular cartilage (n = 1055). Keywords such as repair, hyaluronic acid, and differentiation were also used very frequently. These results indicate that mesenchymal stem cells, PRP applications, and cartilage regeneration are particularly prominent topics in the literature. The most frequently used keywords are presented in Figure 5.

## 4. Discussion

### 4.1. Rapid Growth in Orthobiological Research and the Maturation of the Field

In this bibliometric analysis, it was demonstrated that scientific output on orthobiological treatments in osteoarthritis increased significantly over the last two decades. The acceleration observed in the number of publications, particularly after 2015, suggests that global interest in regenerative medicine and biological therapies is strengthening. The increasing prevalence of osteoarthritis worldwide, the aging population, and the fact that most current treatment options focus on symptom control have increased interest in disease-modifying and regenerative approaches [28,29]. In particular, PRP, mesenchymal stem cells, bone marrow aspirate concentrate, and, in recent years, exosome-based applications have become the subject of intense research due to their potential to restore joint homeostasis and to slow cartilage degeneration.

The increase in the number of publications identified in the present study is consistent with the results of other bibliometric studies in the field of osteoarthritis. Chen et al. (2022), who investigated the relationship between meniscus and osteoarthritis, reported that there was a significant increase in publications in osteoarthritis research over the past decade [30]. Similarly, bibliometric analyses conducted in the fields of physical activity [31], physical therapy applications [32], acupuncture treatments [33], and high tibial osteotomy [34] in knee osteoarthritis also indicate that there is a steady increase in scientific output in osteoarthritis research, indicating that osteoarthritis has transformed into a multidisciplinary research field including not only clinical aspects but also basic science, rehabilitation, bioengineering, and regenerative medicine. The present findings are also consistent with those of previous bibliometric studies focusing on specific orthobiological therapies, such as platelet-rich plasma and mesenchymal stem cells, which similarly reported substantial growth in publication output and increasing global scientific interest over the last decade.

The results obtained in the present study also reflect the maturation process of orthobiological studies. In early studies, the focus was primarily on cell culture, cartilage biology, and experimental regeneration models; however, a significant increase in the number of randomized controlled trials, systematic reviews, and meta-analyses has been observed in subsequent years. This transformation indicates that orthobiological treatments are shifting from the realm of experimental research to practices increasingly integrated into clinical practice and are beginning to be evaluated within the framework of evidence-based medicine. This evolution also reflects an important shift in research priorities. While early investigations primarily focused on demonstrating the biological potential of individual orthobiological products, in recent studies, comparative effectiveness, standardized preparation protocols, patient selection, and long-term clinical outcomes are increasingly emphasized. These developments are essential for translating promising laboratory findings into routine clinical practice and for establishing evidence-based recommendations for the management of osteoarthritis. As the field continues to evolve, greater international collaboration, multicenter randomized controlled trials, and harmonization of methodological standards will be crucial to accelerate the clinical translation of emerging orthobiological therapies.

### 4.2. The Focus of Research Is Shifting from Stem Cells to Exosomes and Next-Generation Biological Therapies

One of the most striking results of the present study is the significant shift in the focus of research over time. In early trend analysis, the emphasis was on cell-based approaches such as bone marrow stromal cells, chondrogenesis, cartilage repair, and mesenchymal stem cells; however, in recent years, exosomes, extracellular vesicles, precision medicine applications, and next-generation drug delivery systems have emerged as prominent research fields. This change demonstrates that orthobiological therapies are evolving from a purely cellular-focused approach towards more complex and bioengineered solutions.

Although mesenchymal stem cells have been at the center of regenerative therapies in osteoarthritis for many years, in recent years it has been discovered that the therapeutic effects of these cells are largely mediated by the biological products they secrete, which has led to a shift in research interest to exosomes and extracellular vesicles. Since exosomes regulate intercellular communication, modulate the immune response, suppress inflammation, and support cartilage homeostasis, they are considered promising biological agents in the treatment of osteoarthritis [35,36,37]. In several experimental studies, it has been demonstrated that mesenchymal stem cell-derived exosomes can reduce inflammation, restore extracellular matrix balance, and promote regenerative processes in osteoarthritic cartilage [38,39]. In recent years, modified or target-designed exosome systems have been developed with gene editing technologies, which has further increased the research momentum in this field [40]. As determined in the present study, exosomes and extracellular vesicles are among the fastest rising research themes in recent years, which is consistent with the transformation from cell-based approaches to cell-free biological products in osteoarthritis research. In current reviews, it is reported that exosomes are considered not only a biological agent in the treatment of osteoarthritis, but also as one of the important components of future precise and personalized treatment strategies [37,41].

However, recently, it has been observed that not only biological agents but also advanced drug delivery systems that enable the effective and controlled delivery of these agents within the joint have gained importance in the treatment of osteoarthritis. It is reported that there is a rapid increase in studies conducted on intra-articular drug delivery systems, nanomaterials, stimulus-sensitive release platforms, and hydrogel-based biomaterials [42,43,44]. Similarly, since hydrogel-based microspheres and biomaterial-supported drug delivery systems are among the research fields of the future, they have the potential to provide controlled release of therapeutic agents and to support cartilage repair [45,46]. Co-consideration of these results suggests that orthobiological research in osteoarthritis is evolving from cell-based regenerative approaches to cell-free biological products, smart biomaterials, and targeted drug delivery systems. In the future, exosome technologies, biomaterial-assisted applications, and personalized treatment strategies are expected to be key focuses of osteoarthritis research. The transition toward cell-free therapies and biomaterial-assisted delivery systems also reflects an important shift in the translational landscape of orthobiological research. Compared with cell-based therapies, exosome-based approaches offer several potential advantages, including lower immunogenicity, easier storage and transportation, reduced regulatory complexity, and greater scalability for clinical application. Nevertheless, several challenges remain before these technologies can be widely adopted in routine clinical practice, including the standardization of exosome isolation and characterization methods, optimization of dosing strategies, quality control, and the generation of robust long-term clinical evidence. Addressing these challenges will be essential for translating these promising experimental advances into safe and effective treatments for patients with osteoarthritis.

### 4.3. China’s Rise in Scientific Production and Global Leadership

In the present study, it was determined that China was by far the leading country in terms of the number of publications. This situation can be explained by the investments made by the Chinese government in biotechnology, stem cell research, and regenerative medicine in recent years. It is known that, especially in the last decade, China has become one of the world’s fastest-growing research ecosystems in the fields of regenerative medicine, biomaterials, tissue engineering, and cellular therapies. As determined in the present study, China-based universities such as Sichuan University, Shanghai Jiao Tong University, and Zhejiang University are among the most prolific institutions, which supports this result.

The results obtained in the present study are consistent with those obtained in other bibliometric studies conducted in the field of osteoarthritis. It has been reported that China is among the most prolific countries in high tibial osteotomy [34], physical therapy applications [32], meniscus and osteoarthritis research [30], and physical activity studies [31], which is not unique to orthobiological treatments but reflects a global trend observed in osteoarthritis research in general.

However, as demonstrated in the present study, China takes the lead in terms of the number of publications, but the United States has a higher impact factor in terms of average citations per article, which highlights China’s quantitative superiority in research output, and suggests that the United States maintains its capacity to produce high-impact publications. A similar situation has been reported in bibliometric studies conducted in different fields of biomedical research. Therefore, it can be predicted that the production of knowledge regarding orthobiological treatments in osteoarthritis will continue to be largely shaped by research networks centered in China and the United States in the future. These findings also highlight the complementary roles of research productivity and scientific influence in advancing the field. While China’s remarkable publication output demonstrates its strong research capacity and sustained investment in orthobiological science, the consistently high citation impact of publications from the United States suggests continued leadership in generating influential and practice-changing evidence. Strengthening international collaboration between these leading research communities may accelerate knowledge exchange, improve methodological quality, and facilitate the translation of innovative orthobiological therapies into clinical practice worldwide.

### 4.4. The Dominant Position of Mesenchymal Stem Cells and PRP on the Research Agenda

The analysis of keywords revealed that mesenchymal stem cells and platelet-rich plasma were among the terms used most frequently, indicating that current orthobiological treatment research is largely shaped around cellular and biological regeneration strategies. Mesenchymal stem cells have become a key focus of research on osteoarthritis due to their differentiation potential, immunomodulatory properties, and tissue regeneration-promoting effects. In their bibliometric analysis, in which they investigated MSC applications in orthopedics (2022), Deng et al. reported that there was a significant increase in the number of publications in this field over the last twenty years, and that MSCs became one of the most important fields of study in orthopedic research [47].

Similarly, PRP stands out as one of the most widely researched orthobiological applications in osteoarthritis because it is easy to apply and has an autologous origin and a rich growth factor content. In their bibliometric analysis (2023), Xiao et al. demonstrated that PRP received increasing attention in knee osteoarthritis and that it was one of the fastest-growing research topics in the field [48]. In their bibliometric study in which they investigated effects of intraarticular injection treatments in knee osteoarthritis (2023), Lu et al. reported that PRP, hyaluronic acid, and stem cell applications held a dominant position in the research agenda [49].

One of the leading factors contributing to this trend is that MSC and PRP applications have promising results in terms of pain control, functional recovery, and biological regeneration in osteoarthritis. As demonstrated in a recently published umbrella review, both MSC and PRP applications can provide clinical benefit in knee osteoarthritis, and existing systematic reviews report generally positive results [50]. Similarly, in their meta-analysis (2024), Aixirefu et al. revealed that MSC and PRP treatments could provide significant improvements in pain and functional outcomes [51]. However, in available evidence, it is also emphasized that results should be interpreted with caution due to heterogeneity in treatment protocols, differences in preparations, and variability in patient selection criteria.

In the present study, MSC and PRP were among the most frequently used keywords, indicating that these two treatments, which form the bridge between basic science and clinical application in orthobiological research in osteoarthritis, are at the intellectual core of the field. However, PRP-derived exosomes, extracellular vesicles, and bioengineered regenerative approaches have emerged in recent years, which [37,41,44,52] suggests that the focus in research is increasingly shifting towards more advanced biological platforms. The central position of MSCs and PRP in the keyword network also reflects their role as benchmark therapies against which emerging orthobiological interventions are increasingly being compared. Rather than replacing these established approaches, next-generation therapies such as exosome-based products and biomaterial-assisted regenerative platforms are expected to complement or enhance their therapeutic potential. Future research should therefore focus on well-designed comparative clinical trials, standardized preparation and reporting protocols, and long-term follow-up studies to determine the relative effectiveness, safety, and cost-effectiveness of these evolving orthobiological strategies.

### 4.5. Thematic Structure and Future Direction of the Field

Thematic map analysis revealed that mesenchymal stem cells and cartilage regeneration constituted the basic knowledge base of orthobiological research in osteoarthritis. This finding is consistent with those of bibliometric studies in which MSC-based therapies are reported to have occupied a central position in both basic science and clinical research over the past two decades [47]. Due to their immunomodulatory effects, paracrine signaling properties, and potential to support tissue repair, mesenchymal stem cells are still among the biological approaches researched most intensively in the treatment of osteoarthritis [37]. On the other hand, the high density and level of sophistication of the PRP and knee osteoarthritis clusters suggest that these areas have become conceptually mature research topics that are strongly integrated into clinical practice. In bibliometric analyses conducted in recent years, it has been demonstrated that PRP is one of the fastest-growing areas of research in knee osteoarthritis and preserves its dominant position among intra-articular injection treatments [48,49]. In current systematic reviews and umbrella review studies, it has also been reported that PRP and MSC applications can provide positive effects on pain and functional results in osteoarthritis [50,53].

The combined evaluation of thematic mapping and trend analysis provides important clues regarding the future direction of the research field. In the present study, exosomes, extracellular vesicles, and next-generation biological products were identified as emerging research themes, which indicates that osteoarthritis research is evolving from traditional cell-based approaches to cell-free biological products. As reported in recent studies, MSC-derived exosomes may play a significant role in regulating inflammation, maintaining cartilage homeostasis, and supporting tissue regeneration [36,37]. However, in the current literature, it is indicated that in future research, the focus will not only be on the development of biological agents but also on their efficient and controlled delivery into the joint. Smart drug delivery systems, stimulus-responsive release platforms, nanotechnology applications, and hydrogel-based biomaterials have stood out as rapidly growing research areas in recent years [43,44]. Hydrogel-supported carrier systems are expected to become an important component of orthobiological treatments in the future, particularly due to their ability to enhance the stability of biological agents and to support cartilage repair [45,46]. These findings are consistent with the broader trend observed across recent bibliometric studies, which have identified a growing shift from conventional regenerative approaches toward cell-free therapies, biomaterial-assisted platforms, and precision medicine in musculoskeletal research.

Considering these findings together, orthobiological research in osteoarthritis is evolving from traditional approaches centered on MSCs and PRP toward advanced strategies involving exosomes, biomaterials, controlled drug delivery systems, and personalized regenerative medicine. Future progress in the field will depend not only on the development of novel biological therapies but also on the establishment of standardized manufacturing protocols, validated biomarkers for patient selection, and high-quality multicenter randomized controlled trials. Furthermore, integrating bioengineering, molecular biology, and artificial intelligence into orthobiological research may facilitate the development of more precise, individualized treatment strategies and accelerate the translation of experimental discoveries into routine clinical practice.

### 4.6. Strengths and Limitations of the Study

The present study has several important strengths. To our knowledge, this is one of the first comprehensive bibliometric analyses to evaluate the global scientific landscape of orthobiological treatments for osteoarthritis by integrating a broad range of therapeutic approaches, including platelet-rich plasma, mesenchymal stem cells, bone marrow aspirate concentrate, stromal vascular fraction, exosomes, and extracellular vesicles, within a single analytical framework. The inclusion of a large dataset comprising 7875 publications enhances the robustness and representativeness of the findings. Moreover, the combined evaluation of publication trends, countries, institutions, authors, collaboration networks, keyword co-occurrence, and thematic evolution provides a comprehensive overview of the development of the field and enables the identification of emerging research directions.

Nevertheless, several limitations should be acknowledged. First, the analysis was restricted to the Web of Science Core Collection (WoSCC). Although WoSCC is widely regarded as one of the most comprehensive and standardized databases for bibliometric studies, publications indexed exclusively in other databases, such as Scopus, PubMed, or Dimensions, were not included, which may have introduced database selection bias. Second, only English-language Articles and Review Articles were included, potentially resulting in language bias and the exclusion of relevant studies published in other languages or document types. Third, citation-based indicators are inherently susceptible to citation bias because older publications have had more time to accumulate citations than recently published studies, and citation counts may also be influenced by factors other than scientific quality. Fourth, because the literature search was conducted on 3 June 2026, publication data for 2026 represent only a partial publication year; therefore, publication trends for that year should be interpreted with caution. Finally, bibliometric analyses describe research productivity, collaboration patterns, and knowledge structures but do not directly evaluate the methodological quality, level of evidence, or clinical effectiveness of the included studies.

In addition, the present analysis was based on conventional bibliometric indicators and did not incorporate normalized or correction parameters (e.g., population-adjusted or economy-adjusted publication metrics), which may provide additional insight into regional differences in research productivity. Future bibliometric studies should consider incorporating such indicators to enable more meaningful cross-country comparisons.

## 5. Conclusions

This bibliometric analysis has demonstrated that research into orthobiological treatments for osteoarthritis has grown significantly over the past two decades, and the field has become increasingly multidisciplinary. It was determined that China and the United States were the two countries that lead research in terms of scientific production and impact, and that mesenchymal stem cells and platelet-rich plasma constituted the main research axes of the field. Thematic and trend analysis revealed that the focus in research is shifting from traditional cellular therapies to innovative approaches such as exosomes, extracellular vesicles, biomaterials, and controlled drug release systems.

The findings demonstrate that orthobiological treatments in osteoarthritis reflect not only current research trends but also scientific directions in studies to be conducted in the future. In particular, cell-free biological products, smart biomaterials, and personalized treatment strategies are expected to be the focus of research in the forthcoming years. The present study is expected to contribute to the understanding of the current knowledge structure, research trends, and future research priorities regarding orthobiological treatments in osteoarthritis, and to offer a comprehensive roadmap to researchers and clinicians.

Future research should focus on conducting high-quality multicenter randomized controlled trials, establishing standardized preparation and reporting protocols for orthobiological therapies, and identifying reliable biomarkers to support personalized treatment strategies. In addition, integrating emerging technologies such as extracellular vesicles, biomaterial-assisted delivery systems, and artificial intelligence into regenerative medicine research may further accelerate the clinical translation of orthobiological therapies for osteoarthritis.

## Figures and Tables

**Table 1:** General bibliometric characteristics of publications on orthobiological treatments in osteoarthritis.

Bibliometric Characteristics	Result
Period analyzed	2000–2026
Total number of publications	7875
Number of journals	1254
Total number of references	198,048
Total number of authors	26,934
Number of single-authored publications	120
Number of authors in single-authored publications	97
Number of co-authors per publication	6.82
International co-authorship rate (%)	21.33
Average publication age (years)	5.65
Average number of citations per publication	35.45
Annual growth rate (%)	22.8
Number of author keywords	9397
Number of Keywords Plus	7450
Number of articles	5551
Number of reviews	2324
Database	Web of Science Core Collection
Analysis software	Bibliometrix (version 4.3.2)/Biblioshiny (version 4.3.2)

**Table 2:** The most prolific journals, authors, and institutions regarding orthobiological treatments in osteoarthritis.

Most Prolific Journals	Number of Publications	Most Prolific Authors	Number of Publications	Most Prolific Institutions	Number of Publications
*International Journal of Molecular Sciences*	238	Giuseppe Filardo	87	Sichuan University	347
*Osteoarthritis and Cartilage*	195	Wang Y	81	Shanghai Jiao Tong University	326
*American Journal of Sports Medicine*	140	Liu Y	71	Zhejiang University	305
*Stem Cell Research & Therapy*	123	Wang J	69	University of California System	200
*Arthroscopy*	92	Zhang Y	64	Peking University	189
*Journal of Orthopaedic Research*	89	Sekiya I	57	Harvard University	175
*Frontiers in Bioengineering and Biotechnology*	88	Zhang J	56	Chinese Academy of Sciences	174
*Scientific Reports*	88	De Girolamo L	55	Central South University	167
*Journal of Orthopaedic Surgery and Research*	81	Kon E	54	Taipei Medical University	167
*Stem Cells International*	81	Li J	53	Sun Yat-sen University	164

**Table 3:** Most prolific countries and countries with the most impact regarding orthobiological treatments in osteoarthritis.

Country	Number of Publications	Number of Total Citations	Average Number of Citations Per Article
China	8703	70,316	29.4
United States	4989	60,817	45.6
Italy	1642	16,231	38.6
South Korea	1038	12,626	42.7
Japan	959	8833	33.2
Spain	940	7850	36.0
United Kingdom	921	9111	40.7
Germany	856	8590	37.3
Iran	700	4814	31.1
India	635	3824	22.4

Note: Country publication counts are based on author affiliations. Publications involving international collaboration may be counted in more than one country; therefore, total country counts may exceed the total number of publications analyzed.

**Table 4:** Top 10 most cited publications on orthobiological treatments in osteoarthritis.

Title	Authors	Journal	WoS Citations	Year	Doi	Reference
Articular cartilage repair: basic science and clinical progress. A review of the current status and prospects	Hunziker E.B.	*Osteoarthritis and Cartilage*	1619	2002	10.1053/joca.2002.0801	[19]
Repair and tissue engineering techniques for articular cartilage	Makris EA, Gomoll AH, Malizos KN, Hu JC, Athanasiou KA.	*Nature Reviews Rheumatology*	1061	2015	10.1038/nrrheum.2014.157	[20]
Osteoarthritis	Goldring MB, Goldring SR.	*Journal of Cellular Physiology*	1051	2007	10.1002/jcp.21258	[21]
The role of synovitis in osteoarthritis pathogenesis	Scanzello CR, Goldring SR.	*Bone*	971	2012	10.1016/j.bone.2012.02.012	[22]
Osteoarthritis: pathogenic signaling pathways and therapeutic targets	Yao Q, Wu X, Tao C, Gong W, Chen M, Qu M, Zhong Y, He T, Chen S, Xiao G.	*Signal Transduction and Targeted Therapy*	956	2023	10.1038/s41392-023-01330-w	[1]
Stem cell therapy in a caprine model of osteoarthritis	Murphy JM, Fink DJ, Hunziker EB, Barry FP.	*Arthritis & Rheumatism*	850	2003	10.1002/art.11365	[23]
Inhibition of TGF-β signaling in mesenchymal stem cells of subchondral bone attenuates osteoarthritis	Zhen G, Wen C, Jia X, Li Y, Crane JL, Mears SC et al.	*Nature Medicine*	830	2013	10.1038/nm.3143	[24]
The knee meniscus: structure-function, pathophysiology, current repair techniques, and prospects for regeneration	Makris EA, Hadidi P, Athanasiou KA.	*Biomaterials*	823	2011	10.1016/j.biomaterials.2011.06.037	[25]
MSC exosomes mediate cartilage repair by enhancing proliferation, attenuating apoptosis and modulating immune reactivity	Zhang S, Chuah SJ, Lai RC, Hui JHP, Lim SK, Toh WS.	*Biomaterials*	763	2018	10.1016/j.biomaterials.2017.11.028	[26]
Intra-articular injection of mesenchymal stem cells for the treatment of osteoarthritis of the knee: a proof-of-concept clinical trial	Jo CH, Lee YG, Shin WH, Kim H, Chai JW, Jeong EC et al.	*Stem Cells*	717	2014	10.1002/stem.1634	[27]

## Data Availability

The bibliographic data used in this study were obtained from the Web of Science Core Collection database. The data supporting the findings of this study are available from the corresponding author upon reasonable request, subject to database licensing restrictions.

## Abbreviations

The following abbreviations are used in this manuscript:

BMAC	Bone Marrow Aspirate Concentrate
EVs	Extracellular Vesicles
MCA	Multiple Correspondence Analysis
MSCs	Mesenchymal Stem Cells
OA	Osteoarthritis
PRP	Platelet-Rich Plasma
SVF	Stromal Vascular Fraction
